# The Mediating Role of Tolerance for Psychological Pain in the Relationship Between Different Types of Childhood Traumatic Experiences and Suicidal Ideation

**DOI:** 10.1177/00302228231169148

**Published:** 2023-04-18

**Authors:** Bruna Passos, Rui C. Campos, Cátia Reixa, Ronald R. Holden

**Affiliations:** 1 Department of Psychology, 70989 University of Évora, Évora, Portugal; 2 Department of Psychology, 4257 Queen’s University, Kingston, ON, Canada

**Keywords:** Childhood trauma, suicidal ideation, tolerance for psychological pain

## Abstract

The aim of the present research was to evaluate the mediating effects of facets of the tolerance for psychological pain on the relationship between childhood trauma and suicidal ideation. A sample of 437 community individuals and a sample of 316 college students participated. For the community sample, managing the pain mediated the relationship between childhood trauma, the different types of traumatic experiences and suicidal ideation. In the college sample, managing the pain and enduring the pain mediated the relationship between childhood trauma, the different types of traumatic experiences and suicidal ideation, except for the case of sexual abuse. The present results have potential clinical implications. Mental health professionals should be aware of the long-term consequences of exposure to childhood trauma and need to assess the ability to tolerate psychological pain so as to implement appropriate psychological interventions that help individuals cope with their pain.

## Introduction

Suicide is a major public health problem and is the cause of more than 700,000 deaths per year worldwide (World Health Organization [WHO], 2021). The occurrence of suicide attempts and suicidal ideation is estimated to be considerably more frequent (Holden et al., 2020). Suicidal behaviors are influenced by the interaction of multiple biological, psychological, environmental, and social factors which in combination may contribute to the great difficulty in predicting death by suicide (O’Connor & Nock, 2014). The literature demonstrates that suicidal ideation is an important risk factor for subsequent death by suicide (Klonsky et al., 2016) and is an important public health concern in and of itself, making it essential to understand its causes so as to implement more effective prevention strategies (Liu et al., 2020). In the present research, we aimed to contribute to understanding suicidal ideation by relating it to childhood trauma and tolerance for psychological pain.

### Childhood Trauma and Suicidal Ideation

Childhood trauma is also a major global public health issue (World Health Organization [WHO], 2020). It is estimated that one in seven children suffers from maltreatment (Centers for Disease Control and Prevention, 2021). Childhood trauma is characterized by any act of commission or omission by caregivers that has the potential to cause harm to a child. Acts of commission are deliberate and intentional and are characterized by offensive words or actions. Acts of commission include physical abuse, sexual abuse, and emotional abuse. Alternatively, acts of omission are the failure to meet a child’s basic physical, emotional, and/or educational needs. This includes physical neglect and emotional neglect (Briere, 2002; Leeb et al., 2008).

Childhood trauma has several long-term sequalae (Norman et al., 2012), including suicidal behaviors. Childhood trauma shows a significant relationship to suicidal ideation in clinical and non-clinical samples (Angelakis et al., 2019; Barbosa et al., 2014; Enns et al., 2006; Miller, 2013; Xinyu et al., 2020). However, the specific links of suicidal ideation to different forms of traumatic experiences have been less studied (Bahk et al., 2017). Nevertheless, some studies have shown that different forms of traumatic experiences may be associated with suicidal ideation (Miller et al., 2013). For example, in research by Barbosa et al. (2014) with a community sample of adolescents and adults, suicide risk was found to be 3.1 times higher among participants who experienced physical abuse, 3.4 times higher among participants who experienced sexual abuse, 6.6 times higher among those who experienced emotional abuse, 2.8 times higher in those who experienced physical neglect, 3.7 times higher in those who experienced emotional neglect, compared to participants who had not experienced any type of traumatic experience during childhood.

In a meta-analysis (Angelakis et al., 2019) based on studies with clinical and with non-clinical adult samples and when compared to individuals who did not experience any type of traumatic childhood experience: physical abuse was associated with 2.5 times higher risk of the individual manifesting suicidal ideation; sexual abuse and emotional abuse were associated with a 2.0 times higher risk of an individual presenting suicidal ideation; and physical neglect and emotional neglect were associated with 1.5 times higher risk of the individual manifesting suicidal ideation. In another meta-analysis (Angelakis et al., 2020) of studies conducted with samples of children, adolescents, and young adults up to age 24 years, childhood physical abuse, sexual abuse, and emotional abuse were associated with 2.5 times the risk of an individual exhibiting suicidal ideation compared to participants without childhood trauma experiences. Additionally, sexual abuse was associated with 4.0 times greater risk of an adolescent having a suicidal plan.

Berardelli et al. (2022) investigated the association of different types of traumatic experiences with suicidal ideation in a sample of patients admitted to a psychiatric hospital. Sexual abuse had a direct effect on suicidal ideation. In turn, emotional abuse and emotional neglect were related to suicidal ideation through dissociation and hopelessness as mediators. Physical abuse and physical neglect did not present a significant relationship with suicidal ideation.

### Psychological Pain and Tolerance for Psychological Pain

According to Shneidman (1993; 1996; 1998), suicide occurs not only due to exogenous factors (e.g., childhood trauma) but also due to intrinsic factors such as psychological pain or psychache. Psychache is a special form of psychological pain, that is, an extreme pain associated with negative emotions, such as feelings of guilt, humiliation, fear, panic, loneliness, and helplessness and arises when the individual’s basic psychological needs are not met (Shneidman, 1993; 1998). However, the intensity of psychological pain is insufficient to explain suicidal risk; it is the fact that the pain is perceived by the individual as unbearable that is relevant. This implies that suicide relates to different individual thresholds for tolerating psychological pain (Shneidman, 1985). Any individual, at any given time in life, can experience psychological pain, so in terms of predicting suicide risk, it may be particularly important to consider the ability to tolerate this psychological pain (Landi et al., 2020).

Tolerance for psychological pain corresponds to the ability to manage psychological pain (Meerwijk et al., 2019) and is a central and multifaceted construct in some theoretical perspectives on suicide. Indeed, to assess tolerance for psychological pain, Orbach et al. (2004) developed a questionnaire – the Tolerance for Mental Pain Scale (TMPS) - whose exploratory factor analysis revealed three factors: surfeit of the pain, which corresponds to the ability to put the pain aside, in active attempts to stop or reduce the pain by not allowing it to interrupt one’s daily routines and activities; belief in the ability to cope with the pain, that is, a passive acceptance of pain, accompanied by optimistic beliefs about its disappearance; containing the pain, that is, the ability to contain the pain, which can help the individual to live with it without actively trying to change it. However, this tri-factor structure has been challenged. Meerwijk et al. (2019), using factor analysis, reduced the number of items in the questionnaire, and identified two underlying facets: managing the pain; and enduring the pain. Managing the pain reflects the presence of coping strategies that enable individuals who experience psychological pain to actively stop or reduce it. Enduring the pain represents the existence of passive coping strategies, derived from the belief that the pain will eventually disappear.

Some research suggests that exposure to childhood trauma leads to a psychological experience of distress, which is reflected in psychache and also leads to a decrease in protective thoughts (e.g., optimism, personal confidence, life satisfaction) regarding more severe suicidal behaviors (Spínola et al., 2020). Li et al. (2019) suggested that individuals exposed to traumatic experiences in childhood, specifically emotional abuse, are more likely to experience negative emotions and may become more vulnerable to experiencing psychache (Klonsky & May, 2015). Further, individuals who had experienced childhood trauma may develop deficits in emotion regulation, expressed in the lack of ability to tolerate psychological pain (Martins et al., 2021).

### Psychological Pain, Tolerance for Psychological Pain and Suicidal Ideation

The relationship of psychological pain to suicidal ideation has been empirically confirmed in several studies with clinical and non-clinical samples. Results have demonstrated a strong, significant association between the two constructs (e.g., Campos et al., 2018; Cheng et al., 2021; Lambert et al., 2020; Verrocchio et al., 2016). In a meta-analysis conducted by Ducasse et al. (2017), the level of psychological pain was higher in individuals who had suicidal ideation and previous suicide attempts as compared to individuals who did not have suicidal ideation or a history of previous attempts. These results also suggested that the relationship between psychological pain and suicidality is independent of the presence of psychiatric disorders.

Evidence has demonstrated that a higher tolerance for psychological pain is associated with lower levels of psychological pain, depression, and suicidal ideation (Becker et al., 2018; Landi, 2020; Levinger et al., 2016). In alignment with this, individuals who have attempted suicide have lower tolerance for psychological pain when compared to individuals who have never attempted suicide (Meerwijk et al., 2019; Meerwijk & Weiss, 2018). Regarding the two dimensions of tolerance for psychological pain, managing the pain may be more important for suicidal risk and specifically for suicidal ideation when compared to enduring the pain (see Landi et al., 2020; Martins et al., 2021), probably because managing the pain involves active coping strategies, as opposed to more passive coping strategies of enduring the pain.

Some research has also indicated that psychological pain may mediate the relationship between childhood trauma and suicidal behaviors (Berardelli et al., 2022), in particular suicidal ideation. Zarrati et al., (2019), in a study of college students, concluded that psychological pain had a mediating role in the relationship between childhood trauma and suicidal ideation. Further, for a clinical sample consisting of patients diagnosed with a major depressive disorder, psychache was shown to have a mediating role in the relationship between childhood trauma and suicide attempts (Demirkol et al., 2020). This mediation was full for physical abuse, emotional abuse, and emotional neglect, and was partial for sexual abuse and physical neglect.

However, research regarding the mediating role of tolerance for psychological pain in the relationship between childhood trauma and suicidal ideation is, to our knowledge, scarce, probably because most research has focused on the experience and intensity of psychological pain, rather than on ways to cope with this pain. Nonetheless, in a study with a sample of patients with a substance use disorder, lower levels of managing the pain were found to fully mediate the relationship between childhood trauma and suicidal ideation, suggesting that traumatic childhood experiences may lead to deficits in emotion regulation, which may contribute, in turn, to increased suicidal ideation (Martins et al., 2021). However, in that research, mediation effects were not tested specifically for the different types of traumatic experiences, that is, childhood trauma was assessed globally.

### Aims of the Study

**Figure 1.**
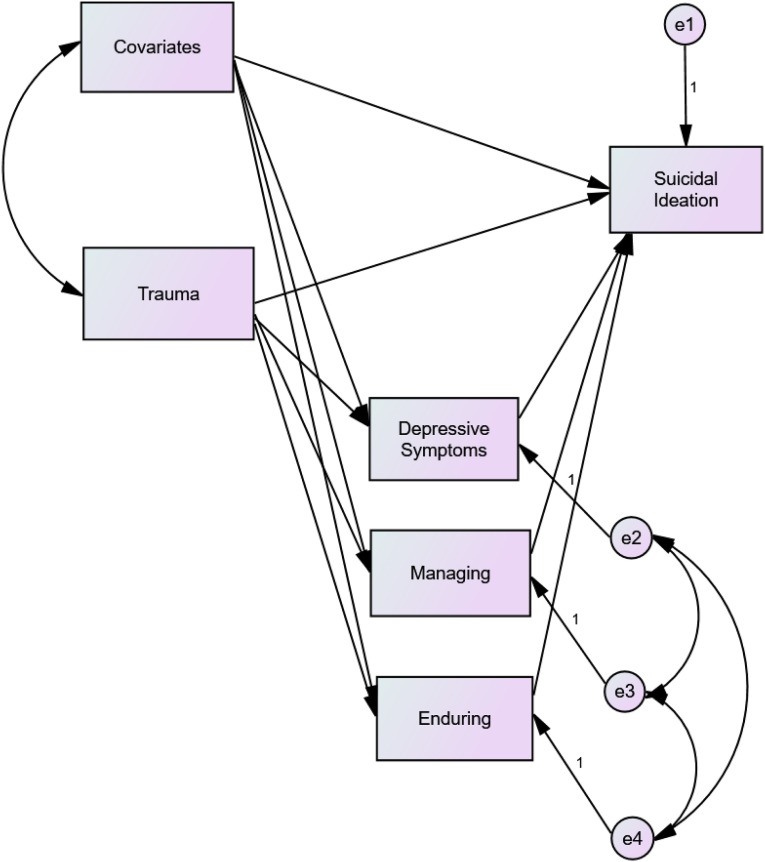
Graphical representation of the tested models.

Suicidal ideation is an important risk factor for subsequent death by suicide and, therefore, it is essential to understand its causes (Rabasco & Andover, 2020). Childhood trauma has been highlighted in the literature as a risk factor for suicidal ideation (Angelakis, et al., 2019). Research has also shown that a low tolerance for psychological pain, in particular managing the pain, demonstrated a mediating role in the relationship between globally assessed childhood trauma and suicidal ideation (Martins et al., 2021). Thus, the aim of the present study was to evaluate, in two samples (a community sample and a sample of college students, chosen by the authors’ convenience), the relationships between various forms of childhood traumatic experiences and suicidal ideation, and the mediating role of the two facets of tolerance for psychological pain (managing the pain and enduring the pain). The effect of depressive symptoms was statistically controlled because depression can be an important consequence of childhood trauma (Gardner et al., 2019; Negele et al., 2015) and is a well-known risk factor for suicidal ideation (Bae et al., 2013; Farabaugh et al., 2012). Additionally, it is a state variable of distress, so controlling for depression permitted us to evaluate the mediating effect of the more stable variable of tolerance for psychological pain, regardless of current symptom severity. The effects of any significant sociodemographic variable were also statistically controlled for. We hypothesized that globally assessed childhood trauma would have a positive and direct relationship with suicidal ideation, as would each one of the various experiences of childhood traumatic experiences: physical abuse; sexual abuse; emotional abuse; physical neglect; emotional neglect. We also hypothesized that lower levels of tolerance for psychological pain (i.e., managing the pain) would mediate the relationship between childhood trauma (considered globally, and within each one of the types of childhood traumatic experience) and suicidal ideation (see Figure 1).

## Method

### Participants

A community sample (Sample 1) and a sample of college students (Sample 2) participated. Sample 1 consisted of 437 participants, 297 women and 140 men, aged 18–65 years (*M* = 43.25; *SD* = 12.61) in which 131 (30%) individuals were single, 66 (15.1%) were cohabiting, 179 (41%) were married, 49 (11.2%) were divorced, and 12 (2.7%) were widowed. Of the 437 participants, nine had a grade 9 education (2.1%), seven had a grade 11 education (1.6%), 77 had a grade 12 education (17.6%), 57 had an undergraduate degree (13%), 261 had a master’s degree (59.7%), and 26 had a doctoral degree (5.9%). In addition, 52 (11.9%) individuals were unemployed.

Sample 2 was composed of 316 participants, 234 women and 82 men aged 18–25 years (*M* = 19.63; *SD* = 1.77) who were attending an undergraduate course at a university. Of the 316 participants, 150 students were in their first year, 83 students were in their second year, and 83 students were in their third year. Of this total sample, 287 were non-working students (91.5%) and 264 (83.5%) had moved away from their homes to study at university.

### Measures

*Sociodemographic forms*. Sample 1’s sociodemographic form consisted of questions about gender, age, education, employment status, occupation, marital status, existence of a chronic illness and/or the presence of a psychiatric disorder diagnosis. For Sample 2, the sociodemographic form inquired about gender, age, year of university course attendance and course being taken, employment, whether he/she was away from home, existence of a chronic illness and/or presence of a of psychiatric disorder diagnosis.

*Childhood Trauma Questionnaire – Short Form (CTQ-SF)* (Bernstein et al., 2003)*.* The CTQ-SF is a self-report measure composed of 28 items which assess the occurrence of maltreatment during childhood, and is a shortened version of the original 70-item Childhood Trauma Questionnaire (Bernstein et al., 1994). This instrument allows for the assessment of the individual’s exposure to five types of traumatic experiences: physical abuse, sexual abuse, emotional abuse, physical neglect, and emotional neglect, which correspond to the five subscales of the questionnaire. Items are rated from 1 (“Never true”) to 5 (“Very often true”). Items 2, 5, 7, 13, 19, 26, and 28 are reverse-keyed because they assess pleasant experiences during childhood. The original version of the CTQ-SF has shown good internal consistency in a community sample (Bernstein et al., 2003), with Cronbach’s alpha coefficients ranging from .61 for the physical neglect subscale to .92 for the sexual abuse subscale. In previous research with a community sample, the Portuguese version (Dias et al., 2013) used in the present research, showed a Cronbach’s alpha coefficient of .84 for the total scale. In the present study, Cronbach’s alpha for the total scale was .81 for each of Samples 1 and 2.

*Center for Epidemiologic Studies Depression Scale (CES-D)* (Radloff, 1977). The CES-D is a self-report measure composed of 20 items that assesses the occurrence of depressive symptoms in the week prior to the assessment. Items are answered using 4-point Likert ratings ranging from 0 “Never or very rarely (less than 1 day)” to 3 “Very often or always (5–7 days).“ Items 4, 8, 12, and 16 are reverse-keyed. The total score of the CES-D is obtained by summing the value of all items and ranges from 0 to 60, with the higher scores indicating greater frequency of depressive symptoms. Initial studies of the scale (Radloff, 1977) indicated strong internal consistency with Cronbach’s alpha coefficient of .85 in three community samples and .90 in a clinical sample. The Portuguese version was developed by Gonçalves and Fagulha (2004). For its scores’ internal consistency, Gonçalves and Fagulha (2004) reported Cronbach’s alpha coefficients of .92 in a sample of college students, .89 in a clinical sample, and .87 in a community sample. In the present study, Cronbach’s alpha coefficients were .93 and .94 in Samples 1 and 2, respectively.

*Tolerance for Mental Pain Scale - 10 (TMPS-10)* (Meerwijk et al., 2019). The TMPS-10 is a 10-item self-report scale, which is a short form of the original 20-item version developed by Orbach et al. (2004). The TMPS-10 scale assesses an individual’s tolerance for psychological pain using two scales: managing the pain and enduring the pain. Items are answered on Likert ratings ranging from 1 (Not at all true) to 5 (Very true). Items 2, 3, 5, 7, and 10 are reverse-keyed. In studies conducted by Meerwijk et al. (2019), high internal consistency was found in a community sample with Cronbach’s alpha coefficients of .90 for managing the pain, and .84 for enduring the pain. The Portuguese version of the original TMPS, prepared by Campos et al. (2019), showed Cronbach’s alpha coefficients of .92 for surfeit of the pain, .75 for belief in the ability to cope with the pain, and .54 for containing the pain, in a university student sample. Also, using the short form with a Portuguese clinical sample (Martins et al., 2021), Cronbach’s alpha coefficient was .87 for managing the pain and .88 for enduring the pain subscales. In the present study, for Sample 1, the internal consistency was .89 for managing the pain and .91 for enduring the pain subscales. For Sample 2, the internal consistencies were .87 for managing the pain and .85 for enduring the pain subscales.

*Suicide Ideation Scale (SIS)* (Luxton et al., 2011; Rudd, 1989). The SIS is a 10-item self-report instrument that measures suicidal ideation in the week prior to the assessment. Items are answered on 5-point Likert ratings, ranging from 1 (Never) to 5 (Always). Scale scores range from 10 to 50, with higher scores representing higher suicidal ideation. Initial studies (Rudd, 1989) showed good internal consistency, with Cronbach’s alpha of .86 for the total scale in a sample of college students, and .91 in a clinical sample (Luxton et al., 2011). The Portuguese version of the scale, developed by Campos et al. (2019), has had a scale score Cronbach’s alpha coefficient of .86 for a community sample. It should be noted that this Portuguese version, unlike the original form, inquires about the 2 weeks prior to the assessment. In the present study, the Cronbach’s alpha coefficient was .93 for each sample.

### Procedures

For Sample 1, inclusion criteria were: (1) age between 18 and 65 years; (2) Portuguese as the native language; (3) at least 6 years of education. Data collection was carried out online using the LimeSurvey platform. The link to the protocol was shared on social media networks. When participants accessed the platform, they were presented, on a first page, with an informed consent form describing the conditions of participation (voluntary participation, without any financial compensation, procedures and aims of the study, guarantee of anonymity and confidentiality, possibility to interrupt the participation at any time). If the participants agreed to participate, they clicked on “next” and then a sociodemographic form was presented, followed by successive questionnaires on each page.

Data for Sample 2 were collected using similar procedures. Inclusion criteria were: (1) age between 18 and 25 years; (2) attending an undergraduate course at a specific Portuguese university; (3) Portuguese as a first language. The access link was sent to the students' institutional email and the protocol was available for 2 weeks. As with Sample 1, a consent form was presented first and, if the participants agreed, they clicked on “next” and were then presented with the sociodemographic form and six questionnaires, four of which were used in the present study.

The informed consent form for each sample included telephone numbers for those who needed (or wanted) to talk to a mental health professional, in particular the psychological counselling line of the National Health Service. The present research was approved by the ethics committee of a Portuguese university.

### Data Analysis

As preliminary analyses using SPSS version 21, the correlations between the study variables were calculated, as well as the correlations between potential covariates and suicidal ideation. The sociodemographic variables that significantly correlated with suicidal ideation were subsequently used in the tested models. Next, to test whether low levels of tolerance for psychological pain (managing the pain and enduring the pain) mediated the relationship between childhood trauma (assessed globally and as separately according to different of traumatic experiences), and suicidal ideation, controlling for the effect of depressive symptoms, several path analysis models were tested (undertaken with AMOS version 21) for each sample using structural equation modelling: (a) six models without mediating variables, entering childhood trauma (assessed globally, or the five forms of traumatic experiences individually) as the exogenous independent variable and suicidal ideation as the endogenous dependent variable, as well as potential significant covariates as exogenous variables; (b) six mediation models, introducing managing the pain and enduring the pain as mediating variables. Depressive symptoms were always introduced as a mediating covariate. To simplify the presentation, only the values for the models with childhood trauma assessed globally are presented in detail. Given the lack of distributional normality of variables, we used bootstrapping methodology with 1000 samples to build 95% bias-corrected confidence intervals for testing the significance levels of the estimated parameters (Yung & Bentler, 1996).

## Results

### Preliminary Analysis

Correlations between the variables are shown in Table 1. For Sample 1, the sociodemographic variables of marital status (defined with a dichotomous variable, single, widowed, or divorced versus married or cohabiting), *r* = −.22, *p <* .001, level of education, *r* = −.17, *p <* .001, being unemployed, *r* = .10, *p < .05*, having a chronic illness, *r* = .13, *p <* .05, and having a psychiatric diagnosis, *r* = .40, *p <* .001, correlated significantly with suicidal ideation. For Sample 2, only having had a psychiatric diagnosis, *r* = .30, *p <* .001, correlated significantly with suicidal ideation. These variables were entered as covariates in the following models.

**Table 1.** *Means,* Standard Deviations, and Correlations Between Study Variables.

											Sample 1		Sample 2	
Variable	1	2	3	4	5	6	7	8	9	10	*M*	*SD*	*M*	*SD*
1. Childhood trauma	____	.66***	.41***	.89***	.73***	.90***	0.48***	−.35***	−.19	.43***	34.86	10.94	38.91	12.36
2. Physical abuse	.63***	____	.18***	.58***	.36***	.47***	.30***	−.20***	−.04	.23***	5.74	1.86	5.93	2.07
3. Sexual abuse	.55***	.37***	____	.31***	.14***	.22***	.15***	−.14***	.06	.13***	5.48	1.64	5.99	2.67
4. Emotional abuse	.89***	.50***	.34***	____	.52***	.71***	.49***	−.37***	.04	.44***	7.94	3.78	9.92	4.54
5. Physical neglect	.64***	.29***	.15***	.48***	____	.61***	.30***	−.23***	−.06	.34***	6.39	2.40	6.36	1.97
6. Emotional neglect	.89***	.39***	.32***	.73***	.56***	____	.42***	−.29***	−.06	.35***	9.29	4.39	10.70	4.82
7. Depression	.42***	.17***	.10	.45***	.22***	.42***	____	−.64***	−05	.60***	16.60	11.56	23.92	13.69
8. Managing the pain	−.40***	−.20***	−.14***	−.41***	−.15***	−.40***	−.38***	____	−12***	−.51***	4.16	0.89	3.68	0.91
9. Enduring the pain	−.24***	−.16***	−.03	−.23***	−.18***	−.24***	−.38***	.38***	____	−.12*	3.33	1.29	3.43	1.04
10. Suicidal ideation	.48***	.21***	.15***	.51***	.31***	.46***	.60***	−.47***	−.47***	____	12.15	5.13	15.40	7.68

*Note.* *p < .05. **p < .01. ***p < .001. Above the diagonal are the values for the community sample (Sample 1; *N* = 437) and below the diagonal are the values for the college student sample (Sample 2; *N* = 316).

### Mediation Models

In the model without mediating variables, in Sample 1, childhood trauma assessed globally had a significant effect on suicidal ideation, controlling for the effect of significant covariates, *β* = .337, *SE* = .053, *p* < .001, 95% CI [0.240, 0.441]. This model explained 32% of the variance. When, instead of trauma considered globally, the experiences of emotional abuse, emotional neglect, sexual abuse, physical abuse, and physical neglect were separately tested, the relationship to suicidal ideation remained significant, with the percentages of variance explained in suicidal ideation being 32%, 29%, 23%, 24%, and 28%, respectively.

In the model without mediating variables, in Sample 2, childhood trauma assessed globally, had a statistically significant effect on suicidal ideation, controlling for the effect of significant covariates, *β* = .435, *SE* = .053, *p <* .005 95% CI [0.322, 0.527]. This model explained 26% of the variance. When, instead of childhood trauma globally, the experiences of emotional abuse, emotional neglect, physical abuse, and physical neglect were separately tested, the relationship with suicidal ideation remained significant, with the percentages of variance explained in suicidal ideation being 29%, 25%, 12%, and 16%, respectively. Note that, in the case of sexual abuse, the relationship was only trending to be significant, *β* = .104, SE = .061, *p <* .10, 95% CI [−0.004, 0.234].

For Sample 1, in a model in which depressive symptoms, managing the pain, and enduring the pain were introduced as mediators, trauma considered globally significantly related to suicidal ideation, *β* = .155, *SE* = .052, *p <* .005, 95% CI [0.053, 0.248], trauma related to managing the pain, *β* = −.288, *SE* = .050, *p* < .001, 95% CI [−0.388, −0.117], and managing the pain related to suicidal ideation, *β* = −.201, *SE* = .052, *p <* .005, 95% CI [−0.292, −0.096]. A significant indirect effect of trauma on suicidal ideation was also found, *β* = .182, *SE* = .029, *p <* .005, 95% CI [0.125, 0.238]. Trauma was unrelated to enduring the pain. The model explained 48% of the variance in suicidal ideation. When considering the different forms of traumatic experiences separately, the results were similar, with the models explaining 48%, 47%, 46%, 46%, and 48% of suicidal ideation when considering emotional abuse, emotional neglect, sexual abuse, physical abuse, and physical neglect, respectively. However, in the case of sexual abuse and physical abuse, the relationship with suicidal ideation was no longer significant. Thus, controlling for depressive symptoms, these results show a partial mediating effect of managing the pain on the relationship of each of global trauma, emotional abuse, emotional neglect, and physical neglect with suicidal ideation, and a full mediating effect on the relationship of sexual abuse and physical abuse with suicidal ideation (see Table 2).

**Table 2.** Summary of the Results of the Mediation Models Tested.

Community Sample			College Student Sample		
Experience of trauma	Mediating effect	Significant mediators	Experience of trauma	Mediating effect	Significant mediators
Global	Partial	Managing pain	Global	Partial	Managing and Enduring pain
Physical abuse	Total	Managing pain	Physical abuse	Total	Managing and Enduring pain
Sexual abuse	Total	Managing pain	Sexual abuse	No effect	-
Emotional abuse	Partial	Managing pain	Emotional abuse	Partial	Managing and Enduring pain
Physical neglect	Partial	Managing pain	Physical neglect	Partial	Managing and Enduring pain
Emotional neglect	Partial	Managing pain	Emotional neglect	Partial	Managing and Enduring pain

For Sample 2, in a model in which depressive symptoms, managing the pain, and enduring the pain were entered as mediators, childhood trauma related significantly to suicidal ideation, *β* = .216, *SE* = .047, *p <* .005, 95% CI [0.122, 0.309]. Trauma also related to managing the pain, *β* = .−345, *SE* = .050, *p <* .005, 95% CI [−0.441, −0.245], and to enduring the pain, *β* = −.206, *SE* = .058, *p <* .005, 95% CI [−0.317, −0.092]. Managing the pain related to suicidal ideation, *β* = .200, *SE* = .060, *p <* .001, 95% CI [−0.320, −0.088], as did enduring the pain, *β* = . 228, *SE* = .061, *p <* .005, 95% CI [−0.308, −0.146]. A significant indirect effect of trauma on suicidal ideation was also found, *β* = .219, *SE* = .034, *p <* .005, 95% CI [0.156, 0.291]. The model explained 51% of the variance in suicidal ideation. When considering the different traumatic experiences separately, the results were similar, with the models explaining 52%, 50%, 48%, and 50% of suicidal ideation when considering emotional abuse, emotional neglect, physical abuse, and physical neglect, respectively. Because sexual abuse did not relate to the mediating variables, it was not possible to obtain a significant mediation effect, and in the case of physical abuse, the relationship with suicidal ideation was no longer significant. Thus, controlling for depressive symptoms, these results show a partial mediation effect of managing the pain and enduring the pain in the relationship of childhood trauma, emotional abuse, emotional neglect, and physical neglect with suicidal ideation, and a total mediation effect in the relationship between physical abuse and suicidal ideation (see Table 2).

## Discussion

The present research examined the effect of childhood trauma on suicidal ideation, as well as the mediating role of dimensions of tolerance for psychological pain (managing the pain and enduring the pain) in the relationship between those two constructs, controlling for the influence of depressive symptoms as a mediating factor and for the sociodemographic variables that significantly correlated with suicidal ideation. To demonstrate the robustness of our findings, two distinct samples of community and college students were investigated.

It was hypothesized that global childhood trauma, as well as different subtypes of traumatic experiences would show a positive and direct relationship to suicidal ideation. This hypothesis was partially confirmed. The results in the community sample showed that childhood trauma, considered globally, and each different type of traumatic experiences all demonstrated a significant and direct relationship with suicidal ideation. This result aligns with previous research (e.g., Angelakis et al., 2019; Barbosa et al., 2014; Enns et al., 2006; Miller et al., 2013; Xinyu et al., 2020), that systematically showed a significant relationship between trauma and suicidal ideation. In our college student sample, childhood trauma globally, physical abuse, emotional abuse, physical neglect, and emotional neglect each showed a significant and direct relationship with suicidal ideation, in line with what was hypothesized. In our university sample, however, sexual abuse was only trending to be significantly related to suicidal ideation.

It was also hypothesized that low levels of tolerance for psychological pain (managing the pain) would mediate the relationship of childhood trauma, globally, and each childhood traumatic experience with suicidal ideation. This hypothesis was also partially confirmed. In the community sample, low levels of managing the pain partially mediated the relationship among childhood trauma globally, emotional abuse, physical neglect, and emotional neglect and suicidal ideation, and fully mediated the relationship of physical abuse and sexual abuse with suicidal ideation. This result partially agrees with the findings of Martins et al. (2021) where managing the pain was found to fully mediate the relationship between childhood trauma and suicidal ideation. In our sample of college students, results demonstrated that low levels of managing the pain and enduring the pain partially mediated the association of global childhood trauma, emotional abuse, physical neglect, and emotional neglect with suicidal ideation. Sexual abuse, contrary to what was expected, showed no significant relationship with any of the mediating variables so there was no mediating effect to evaluate. Regarding physical abuse, mediation was full in both samples, indicating that it is a significant traumatic experience that contributes more to lower levels of psychological pain tolerance and, consequently, higher levels of suicidal ideation.

Overall, despite some differences, the results for both samples consistently showed that the link between childhood trauma and suicidal ideation is mediated by some level of tolerance for psychological pain, controlling for depressive state symptomatology. Whereas, for the community sample, managing the pain was the significant mediator, in the college sample both managing the pain and enduring the pain demonstrated significant mediating effects. Further, in the college sample, in contrast to the community sample, sexual abuse did not show a significant relationship with the mediating variables and showed a weak relationship with suicidal ideation. Of note, previous studies have demonstrated the important contribution for sexual abuse in the prediction of suicidal ideation (Bahk et al., 2017).

The absence of a relationship between sexual abuse and the mediating variables and the weak relationship between sexual abuse and suicidal ideation in our sample of college students could have been due to a social desirability effect, associated with the topic being assessed. It is not easy to acknowledge such impactful experiences such as sexual abuse or suicidal ideation. However, the mean score for suicidal ideation was either higher or in a similar range compared to some studies (e.g., Campos et al., 2019; Fitriana et al., 2022) and higher compared to the mean score obtained by the community sample in the present study. This seems to indicate that students did not underreport their painful experiences, namely suicidal ideation. Additionally, the mean scores for sexual abuse in our two samples were similar. It could be that, for a sample of Portuguese college students, individuals who were sexually abused during childhood had received psychological treatment because, in Portugal, college students have more access to psychological services than do individuals in the community. As such, this could explain why sexual abuse does not relate to the mediating variables and has only a weak relationship to suicidal ideation.

We can also speculate that college students in this stage of development who have experienced childhood sexual abuse may manifest their distress through externalizing psychopathology (Lewis et al., 2016), such as risky sexual behaviors (Lalor & McElvaney, 2010), substance or alcohol abuse (Almuneef, 2021), and not in more internalized processes such as psychological pain. We can further postulate that dissociation may hamper the participant’s abilities to respond to self-report items about management of mental pain. If participants distance themselves from their internal experience, their abilities to assess their own coping skills in dealing with pain can be impaired.

The other difference between the samples was that enduring the pain was found as a mediator in students, but not in the community sample. That is, lower levels of managing the pain mediated the relationship between childhood trauma and suicidal ideation in both samples, while lower levels of enduring the pain mediated the relationship between childhood trauma (except sexual abuse) and suicidal ideation, only for the college student sample. To be noted, however, is the nature of the items comprising the enduring the pain scale of the TMPS-10 (e.g., “I believe time will make the pain go away,” “I believe I will find a way to reduce the pain.”). It may be more painful for a younger individual to feel that his/her psychological pain in the future will not change than it is for older individuals. As such, it can be argued that the difference between the samples could be due to different developmental stages and that, over the course of development, the impact of fewer passive strategies of pain tolerance on suicidal ideation may decrease.

### Limitations, Future Directions, and Conclusion

This research has potential limitations. First, a cross-sectional design was used, limiting the drawing of causal inferences (Rindfleisch et al., 2008). For future investigations, it is recommended that the relationships examined here be tested through longitudinal designs. Second, the exclusive use of self-report measures may have led to response biases. The topic under study involves stigma that may have resulted in a bias associated with social desirability (Dowling et al., 2016). This suggests that participants could have felt inhibited in revealing the presence of suicidal ideation or childhood traumatic experiences. To address this potential limitation, investigations using other methods of data collection, such as clinical interviews, are recommended. Third, data were collected online. Online data collection raises some issues such as self-selection and difficulties in generalizing results (Hauser et al., 2018). In addition, the study was conducted with low-risk samples. As such, sampling from other populations (e.g., clinical samples) is recommended to establish the generalizability of current results. Also, both samples, being comprised of volunteers, may not have been fully representative of their respective populations – in the community sample, 68% were women, and 60% had 15 years of schooling while, in the student sample, 74% were women and 47.5% were first-year undergraduates. This may, again, limit the generalizability of obtained results. Thus, it is also recommended that future research be conducted with samples involving childhood sexual abuse. This may assist in the understanding of how, at different stages of development, individuals who have endured this type of abuse express their suffering.

In conclusion, results of the present study indicated that childhood trauma potentiates suicidal ideation, and that low levels of tolerance for psychological pain have an important mediating role in this relationship. Results demonstrated consistency across samples, with managing the pain being a mediating variable. Of note, in students but not in community individuals, childhood trauma leads to the use of both fewer active and passive strategies to manage pain, either of which, in turn, contributes to suicidal ideation. Additionally, in each sample, physical abuse was the traumatic experience that most contributed to a lower ability to tolerate psychological pain which, in turn, contributed to higher levels of suicidal ideation.

The present results have potential clinical implications. First, mental health professionals need to be aware of the long-term consequences, including suicidal ideation, of exposure to childhood trauma in its various forms. Second, professionals, when assessing suicide risk in individuals exposed to childhood trauma, need to assess not only the intensity, but also the ability to tolerate psychological pain so as to implement psychological interventions that take these factors into account, because they contribute to suicidal ideation, a major risk factor for subsequent completed suicide. Interventions should not only focus on psychological pain and its influence on suicidal ideation, but also on helping and empowering the individuals to cope with their pain. Given that psychological pain results from frustrated psychological needs, several psychotherapeutic strategies could be implemented, such as altering the current standing of a client on the psychogenic dimension that is frustrated, adjusting the client-perceived ideal level of the thwarted psychogenic dimension, making the thwarted psychogenic dimension less relevant for the client, or having the client learn to cope with the psychological pain generated by the frustrated psychogenic need.
